# What if … ? A new hypothesis to approach the relationship between environmental stimuli, biological features, and health

**DOI:** 10.1016/j.heliyon.2023.e22985

**Published:** 2023-11-28

**Authors:** Valentina Bollati, Elia Mario Biganzoli, Michele Carugno

**Affiliations:** a EPIGET - Epidemiology, Epigenetics and Toxicology Lab, Department of Clinical Sciences and Community Health, University of Milan, 20122 Milan – Italy; b Epidemiology Unit, Fondazione IRCCS Ca' Granda Ospedale Maggiore Policlinico, 20122 Milan – Italy; c Department of Biomedical and Clinical Sciences (DIBIC), University of Milan, 20157 Milan, Italy; d Data Science Research Center (DSRC), University of Milan, 20157 Milan, Italy

**Keywords:** Epigenetics, DNA methylation, Exposome, Adaptation, Misadaptation

## Abstract

The "exposome" covers all disease determinants across a lifetime. Many exposome factors could induce epigenetic changes, especially in DNA methylation. Yet, the role of these modifications in disease development remains partly understood.

Although the possible relationship among the exposome factors, epigenetic modifications, and health/disease has been investigated extensively, all previous studies start from the assumption that epigenetic changes are always detrimental to (or represent an adverse effect on) the health of the affected individual. We hereby propose a new approach to investigate these modifications, and their possible relation with human health, in the context of the exposome.

Our hypothesis is based on the possibility that some environmentally-induced changes are plastic entities, responding physiologically to the environment to allow individual adaptation. Briefly, after evaluating the association between environmental exposure and the variation of a given biological parameter through regression models, we use the estimated regression function to predict values for each study subject. We then calculated the relative percent difference (PD) between the measured (i.e., observed) biological parameter and the predicted (i.e., expected) from the model. Notably, we have tested our hypothesis using two distinct models, specifically focusing on LINE-1 methylation and extracellular vesicles (EVs). We hypothesize that the greater the difference between the observed and the expected, the greater the inability of the subject to adapt to external stimuli.

## Introduction

We present the following point of view, which stems from our research and observations in the field of environmental exposures and epigenetics. We propose this novel approach with the hope that it will spark interest and discussion among the scientific community. By focusing on the discrepancy between "what should be" and "what is" in terms of an individual's biological response to environmental stimuli, we aim to shed light on the potential adaptive capacity of individuals and its implications for disease risk. We encourage researchers and experts to consider and explore this perspective further, developing novel, more complex methods, as it may offer valuable insights into the multifaceted interplay between epigenetics, environmental exposures, and individual health outcomes.

According to the WHO (World Health Organization), an estimated 24 % of the global disease burden and 23 % of all deaths can be attributed to modifiable environmental factors. Nine million deaths per year (16 % of deaths worldwide) were attributed to air, water, and soil pollution alone [1]. Environmental threats to human health include numerous environmental pollutants including metals, endocrine disruptors, polycyclic aromatic hydrocarbons, dioxin-like chemicals, polychlorinated biphenyls, polyfluoroalkyl substances (PFAS), and several classes of pesticides.

The disease often results from interactions between genes and the environment experienced throughout the life course [2,3]. Environmental exposures, however, cannot be limited to the above-mentioned toxicants, as it is becoming increasingly clear that non-conventional exposures (e.g., stressful events, social life, and parental care) can modulate disease risk.

The awareness that a multitude of factors may synergistically modulate the state of health/disease led to the concept of “exposome,” first defined in 2005 by Chris Wild [4]. The exposome includes the totality of human disease determinants encountered during the life course, and can be divided into three main domains: i) the internal environment (e.g., metabolism, endogenous circulating hormones, morphology, physical activity, gut microflora, inflammation, lipid peroxidation, oxidative stress, and age-related processes); ii) specific external exposures (e.g., radiation, infectious agents, chemical contaminants and environmental pollutants, diet, lifestyle factors, tobacco, alcohol, occupation, and medical interventions); iii) the general external exposome that includes the wider social, economic, and psychological influences on the individual (e.g. social capital, education, financial status, psychological and mental stress, urban-rural environment, and climate) [5].

The aim of the exposomic concept would be to track an individual's exposures (from pre-conception to death) to elucidate how exposures from our environment, diet, and lifestyle interact with our genetic backgrounds.

A growing compendium of evidence indicates that many of the factors listed among those constituting the exposome might induce epigenetic modifications, in particular, modifications of the DNA methylation pattern [6,7]. However, the relative contribution of these modifications to disease development is only partially understood. We hereby propose an alternative approach to investigate these modifications, responding to environmental triggers.

Environmental epigenetics (i.e., the epigenetic pattern shaped by environmental exposures) holds great potential to provide a biomarker that may easily assess and summarize all lifestyle habits and environmental exposures. Although the possible relationship among exposures, epigenetic modifications, and health/disease has been investigated extensively, all previous studies start from the assumption that epigenetic changes are always detrimental to (or represent an adverse effect on) the health status of the affected individual. But … *what if* some of these changes were plastic entities, responding physiologically to the environment to allow individual adaptation?

**Fig. 1:**
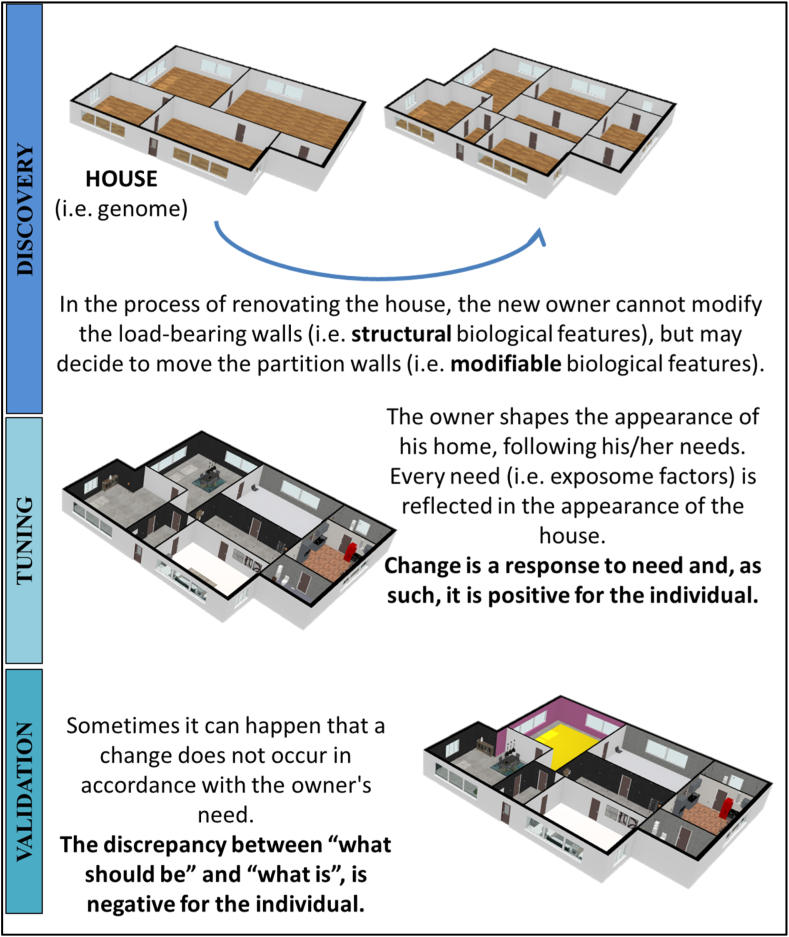
Change is the key to adaptation.

To make a metaphor, we can think of a home acquired by a new owner (Fig. 1): the genome is represented by a house that has just been sold. The new owner can modify the house according to his/her own needs. However, not everything can be changed; load-bearing walls, for example, cannot be moved. The new owner needs are shaped by the exposome and the walls symbolize the epigenetic markers in the genome; the exposome cannot alter completely the epigenetic markers that play a structural role, thus possibly leading to cell/tissue death or major disfunction but can instead determine other epigenetic changes to meet the needs of adaptation to a changed environment. Each new owner's need (i.e., exposome factors) must be reflected in the adaptation of one or more rooms.

Change is a response to a need and, as such, it is expected to be positive for the individual in the homeostasis perspective.

However, some of these changes may not prove to be functional for the needs of the new owner: e.g., a fuchsia room in a predominantly gray-themed house would be out of place. Analogously, an epigenetic modification intended to address a specific environmental factor, which presents a temporary challenge to the individual, might be crucial to restore a new homeostasis and do not clash with the global equilibrium.

The discrepancy between “what should be” (expected on the average) and “what is” (observed on the individual) could be possibly negative for the individual.

So … how could we quantify this “discrepancy”? We hypothesized the following method.

i) After evaluating the dependence between an environmental exposure (e.g., air pollution) and the expected value of a given biological parameter (e.g. conditional mean DNA methylation of a certain sequence or extracellular vesicles count) through a regression model, we estimate predicted values of the biological parameter for each study subject;
ii) We then calculated the percent difference (PD) between the measured (i.e., observed) biological feature and the estimated conditional mean (i.e., expected) from the model, according to the following formula: $PD=\frac{observed-expected}{expected}∙\;100$;
iii) We hypothesize that the greater the difference between the observed and the expected, the greater the “discrepancy” (i.e., potential inability) of the subject to adapt to external stimuli with respect to the average population behavior. This can also be pointed out by selecting those subjects with extreme PD values, i.e., with values above or below arbitrary cut-offs of their distribution (chosen according to the biological parameter distribution), where we observe potential misadaptation.

The decision to use an arbitrary cut-off, instead of using statistical methods such as the z-scores or the use of quantiles, doesn't limit the definition of subjects experiencing misadaptation to a data distribution specific for a given population. However, it requires a quite extended knowledge of the biological feature under exam and its behaviour in reference populations.

According to this point of view, as a proof of concept we applied this approach to two sets of data.

**Fig. 2:**
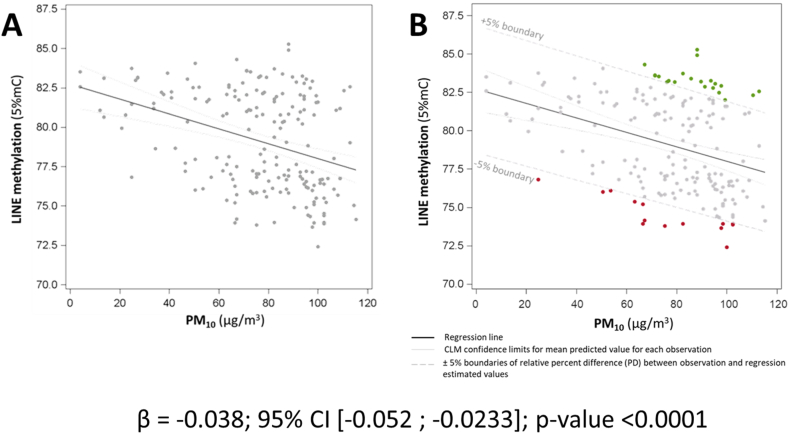
Association between PM_10_ exposure and LINE-1 methylation in the SPHERE study (panel A). Red and green dots represent the subjects showing a percent difference between the measured LINE-1 methylation and the predicted value >|5|% (panel B). (For interpretation of the references to colour in this figure legend, the reader is referred to the Web version of this article.)

First, we used LINE-1 methylation data measured in a subgroup of the SPHERE study, a cohort of subjects established in 2011 (project number: ERC-2011-StG 282413) [8]. LINE-1 is one of the most active DNA retrotransposons in mammals. LINE-1 promoters are usually methylated (and, thus, silenced) in the normal cells [9], but can be “switched on” in response to many environmental triggers [10]. In a randomly selected group of 185 subjects, LINE-1 methylation was assessed by bisulfite-Pyrosequencing, and its association with exposure to particulate matter ≤10 μm (PM10) was evaluated by applying linear regression models. We observed a negative association between PM_10_ (average concentration of the week preceding sample collection) and LINE-1 methylation (β = −0.038; 95 % CI -0.052; −0.0233; p-value <0.0001), in agreement with previous evidence. Applying the new approach that we are proposing, we used the intercept and the regression coefficient to estimate the predicted values of LINE-1 for each study subject (Fig. 2a). We then calculated the PD between the measured LINE-1 methylation values and the estimates predicted from the model. Red dots in Fig. 2b represent subjects showing a negative PD (i.e., observed values lower than estimated ones) higher than 5 %: since predicted values are somewhat representative of what we would “expect” based on the model, we could interpret such a PD as suggestive of the subject's inability to adapt to external stimuli. Green dots, on the other hand, represent subjects showing a positive PD (i.e., observed values greater than predicted/expected ones) higher than 5 %, thus suggestive of the subject's overreaction to external stimuli (i.e., still representing misadaptation). We evaluated the health status of these two groups of subjects and, very interestingly, those with PD below the lower boundary (red dots), are associated with 4-fold greater risk of developing metabolic syndrome when compared to all other subjects (Odds Ratio [OR] = 3.78; 95 % CI 1.28–11.20; p-value = 0.016). Moreover, these subjects had a 9-fold higher risk of hypertension (OR = 8.97; 95 % CI 1.15–69.82; p-value 0.036). We might also speculate that subjects with PD above the higher boundary might have a higher risk of developing other disorders, such as neurodegenerative diseases (which have been previously reported to be associated with a higher LINE-1 methylation). Unfortunately, we don't have data available on this study population to support also this hypothesis as well.

**Fig. 3:**
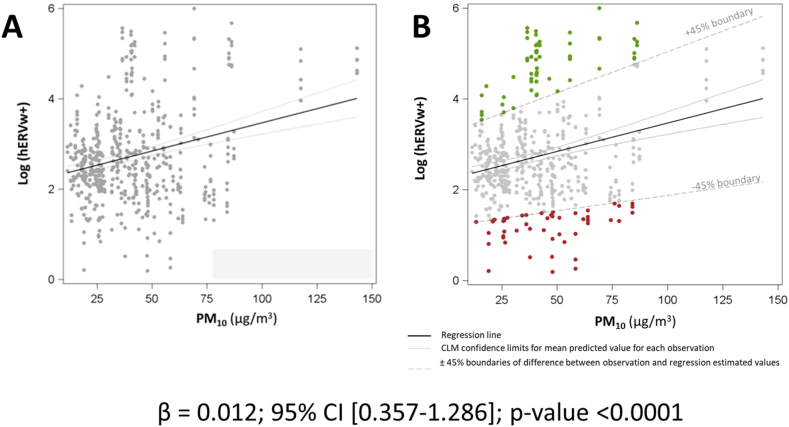
Association between PM_10_ exposure and HERVw-positive extracellular vesicles (EVs) in the INSIDE study (panel A). Red and green dots represent the subjects showing a percent difference between the measured HERVw-positive EVs and the predicted value >|30|% (panel B). (For interpretation of the references to colour in this figure legend, the reader is referred to the Web version of this article.)

Second, we applied the same approach to 518 pregnant women enrolled during the 12th week of gestation within the INSIDE study [11]. The INSIDE study aims to assess the molecular effects of environmental PM exposure during pregnancy, with a particular focus on the role of maternal extracellular vesicles (EVs). EVs are spherical structures limited by a lipid bilayer which can be generated by cells, secreted into the extracellular space, and then enter biological fluids. Although EVs cannot be considered “epigenetics” (if we apply to this word its narrowest definition), they have an important role in mediating the crosstalk between the exposome and potentially all tissues inside the body. Moreover, EVs have a well-known role during pregnancy: syncytiotrophoblast-derived EVs are released in increasing amounts during pathological pregnancy [12] and interact with immune cells modulating them [13]. Remarkably, Syncytin-1 + EVs produced by the placenta can modulate maternal immunity, potentially targeting immune cells to permit fetal development [14]. We observed that PM_10_ levels experienced by mothers on the day preceding the blood drawing were positively associated with Syncytin-1 + EVs (Fig. 3A). Even in this case, women with an “exaggerated” reaction to the environmental noxa (i.e., those with PD > 30 %) (Fig. 3B), had a 6-fold greater risk of developing gestational diabetes than all other subjects (OR = 5.57; 5.57; 95 % CI [1.90–16.29]; p-value 0.0017). Moreover, even considering the discrepancy between the observed and the predicted values of the model (residual of the model) as a continuous variable, we observed a significant association between increasing residual values (i.e., increasing widening of the distance between what we expect and what we observe, reported for unit increments of the residual values) with the value of maternal diastolic blood pressure (Δ% = 8.9 %; 95 % CI 1.2–17.1; p-value 0.02).

Taken as a whole, these results can suggest that subjects who, for any reason, are unable to adapt to environmental stimuli have a higher risk of disease.

According to our point of view, although environmental exposures shape the individual epigenetic pattern, each of us has an intrinsic adaptive capacity that allows us to react to continuous stimuli from the environment. However, in some contexts and particular conditions, this adaptive capacity can decrease.

The concept of adaptive capacity is intimately linked to disease risk. Besides the applicability of this approach to potentially all epidemiological studies involving the study of the effect exerted by an exposome factor on any biological parameter, the application of this new paradigm might have also profound implications in terms of preventive medicine and identification of hypersusceptible subjects. For example, we can imagine a test that works similarly to the Oral Glucose Tolerance Test (OGTT) [15], which is routinely performed during pregnancy to estimate the risk of gestational diabetes mellitus. The OGTT allows for assessing how glycemia levels change after taking a known dose of glucose, i.e., whether the body has a normal or impaired glucose metabolism. Under normal conditions, after a standard oral glucose load, blood glucose levels rise after a few minutes and, within a few hours, glycemia drops to baseline-like levels. If blood glucose does not follow the expected kinetics, diabetes is diagnosed. Similarly, in the case of the new approach we are proposing, it would be possible to verify the measure of a biological feature after stimulation with a trigger, to test the performance of the adaptive response. The relevance in public health would be striking; it would provide an important new tool for the evaluation of individual susceptibility to environmental pressure factors, enabling the development of strategies to prevent pathogenetic processes and consequent tissue and organ damage.

In 2013, Steve Horvath introduced a groundbreaking concept known as the "epigenetic clock" [16], which anticipated the intriguing discrepancy between "what should be" and "what is" in terms of an individual's biological age. This innovative method estimates biological age by analyzing DNA methylation levels at specific CpG sites, which have shown a robust correlation with age. By comparing the predicted biological age using the epigenetic clock with an individual's chronological age, the epigenetic clock assesses the divergence between the actual state of aging at the cellular level and the expected age based on the passage of time.

In the context of epigenetic clocks, a lower predicted value compared to the observed value is considered favorable, implying a healthier biological age than the chronological age. The main distinction between the approach we are depicting through this point of view and the epigenetic clocks lies in the novel way of interpreting the findings, introducing the concept of adaptive capacity in response to environmental stimuli. Instead of solely considering a lower predicted value as favorable, we suggest that some epigenetic changes may represent plastic entities, reflecting an individual's physiological response to the environment and facilitating adaptation. Our approach challenges the traditional notion that all epigenetic changes are inherently detrimental to health. By considering the discrepancy between the observed and expected values as a measure of adaptive capacity, we offer a fresh lens through which to examine the intricate interactions between environmental exposures and epigenetic modifications.

This paradigm shift in the interpretation of epigenetic changes in response to environmental stimuli opens up exciting possibilities for the field of environmental epigenetics. It prompts us to explore the potential functional significance of certain epigenetic modifications and their role in promoting health, rather than just focusing on their association with disease risk.

As we encourage further exploration and validation of our proposed approach, we aspire to inspire a wave of innovative research that not only advances our understanding of the epigenetic effects of environmental exposures but also paves the way for the development of personalized strategies for disease prevention and intervention.

We have to acknowledge that measurement errors in environmental exposure assessment can arise from various sources, such as inaccuracies in data collection methods, laboratory techniques, or the variability of exposure over time. These errors can lead to misclassification of individuals into different exposure groups, potentially introducing bias in our analysis. If the discrepancy between "what should be" and "what is" is primarily driven by measurement error in exposure assessment, it could limit the validity of our model's predictions. To mitigate the impact of potential measurement errors on our results, we might suggest to conduct further analyses in outliers, including the replication of exposure measurements and incorporating multiple time-point measurements where feasible.

We believe that this approach holds the potential for broader applicability. While our current work focused on one specific environmental exposure (i.e., PM exposure), the underlying principles could be extended to encompass various risk and lifestyle factors (e.g., body mass index, smoking). The main challenge will lie in effectively combining these real-life factors to obtain a comprehensive and accurate understanding of an individual's capacity to adapt.

## Funding

The current hypothesis has been formulated based on the generous support of the following grants: 10.13039/501100000781European Research Council, ERC-2011-StG 282413; 10.13039/501100000781European Research Council, ERC-2022-CoG 101086988; and Ministero dell’Istruzione, dell’Università e della Ricerca, PRIN 2015–20152T74ZL.

## Data availability statement

Data will be made available on request.

## CRediT authorship contribution statement

**Valentina Bollati:** Conceptualization, Funding acquisition, Writing – original draft. **Elia Mario Biganzoli:** Conceptualization, Formal analysis, Writing – original draft. **Michele Carugno:** Conceptualization, Formal analysis, Writing – original draft.

## Declaration of competing interest

The authors declare that they have no known competing financial interests or personal relationships that could have appeared to influence the work reported in this paper.

## List of abbreviations used

PD: percent difference
PM10: particulate matter ≤10 μm
EVs: Extracellular Vesicles
OR: Odds Ratio
OGTT: Oral Glucose Tolerance Test
